# Hematological parameters on the effect of the jellyfish venom *Cassiopea andromeda* in animal models

**DOI:** 10.1016/j.dib.2017.02.054

**Published:** 2017-03-09

**Authors:** Iraj Nabipour, Gholamhossein Mohebbi, Hossein Vatanpour, Amir Vazirizadeh

**Affiliations:** a The Persian Gulf Marine Biotechnology Research Center, the Persian Gulf Biomedical Research Center, Bushehr University of Medical Sciences, Bushehr, Iran; b Department of Pharmacology and Toxicology, Faculty of Pharmacy, Shaheed Beheshti Medical Sciences University, Tehran, Iran; c Department of Marine Biotechnology, The Persian Gulf Research and Studies Center, The Persian Gulf University, Bushehr, Iran

**Keywords:** *Cassiopea andromeda*, Crude venom, Hematological effects

## Abstract

For the first time, we previously recorded an enormous population of the *Cassiopea andromeda* jellyfish that had increased dramatically from Bushehr coasts of Iran. The sub-acute toxicity of the jellyfish venom in rat organs was correspondingly carried out. The data presented in this paper relate to the *in vivo* and *in vitro* hematological effects of this venomous species of jellyfish venom.

**Specifications Table**

**Table:** 

Subject area	Toxinology
More specific subject area	Toxinology of jellyfish
Type of data	Figure, Table, Text file
How data was acquired	UV–vis spectrophotometer (Cecil, England), Unicell DXC 800 autoanalyzer (Beckman Coulter, Inc., Fullerton, CA, USA), Sysmex XE-5000 hematology analyzer.
Data format	Analyzed
Experimental factors	The nematocysts and tentacles separations were performed according to Bloom et al.
	The mortality rate was measured, according to Wiltshire et al.
	The haemolysis experiment of crude venom was assessed using human erythrocytes according to the method of Garnier et al.
Experimental features	The chemical analysis of serum electrolytes was analyzed by Unicell DXC 800 autoanalyzer.
	The performance evaluations of hematological parameters were performed using the Sysmex XE-5000 hematology analyzer.
	The measure the amount of RBC lysis and released hemoglobin was determined using a Cecil UV–vis spectrophotometer at 540 nm.
Data source location	Bushehr, Iran, 27° 30′ S; 52° 35′ E
Data accessibility	Data is within this article

**Value of the data**

• The data of the *in vivo* hematological parameters were measured by using the hematology analyzer, and reveal a significant decrease in the levels of RBC, Hb, MCV, MCH, and HCT, as well as, a significant increase in WBC, in testing groups, compared with control groups.
• The data of the *in vitro* hemolytic activity show the venom of the jellyfish was potently hemolytic to human erythrocytes.
• Our observations indicated the necrotizing and hemorrhaging effects of the venom in rats. Evaluation of the hematological parameters together with the clinical manifestations is very helpful for the diagnosis and treatment of the envenomation.
• We release these data so they can be applied in future investigations for their technical aims.

## 1 Data

### 1.1 Hematological study *in vivo* and *in vitro*

The LD_50_ (IV mouse) was estimated at 104.0 µg/kg BW in a 24 h observation period. The significant changes in the levels of some hematological parameters in blood sample of male Wistar rats, comprising the red blood cells (RBC), white blood cells (WBC), hemoglobin concentration (Hb), mean corpuscular volume (MCV), mean corpuscular hemoglobin (MCH) and mean corpuscular hemoglobin concentration (MCHC) and hematocrit (HCT) after administration of sub-lethal doses (1/2 and 1/3 of LD_50_) of jellyfish venom, compared with control group are shown in Table 1.

The *in vitro* hemolytic activities of the venom were 0.36±0.009 and 0.38±0.006 respectively in 1/3 and 1/2 LD_50_ doses. The hemolytic activities of normal saline (NS) and deionized water (DW), as the negative and positive control groups were respectively 0.066±0.0002 and 0.39±0.00. To be precise, the hemolytic activities of the jellyfish venom in 1/3 and 1/2 LD_50_ doses were respectively 92.31 and 97.44% than the deionized water as the positive control group (100% hemolytic activity).

### 1.2 Serum electrolytes

A summary of the results of serum electrolytes, including sodium (Na), potassium (K), calcium (Ca), magnesium (Mg), phosphorus (P) and iron (Fe) and other common blood chemistry comprising serum albumin, creatinine, uric acid, glucose, triglyceride, and haptoglobin are also revealed in Table (1).

### 1.3 The necrotizing and hemorrhaging effects of the venom inducted by Hemolytic activity

**Fig. 1:**
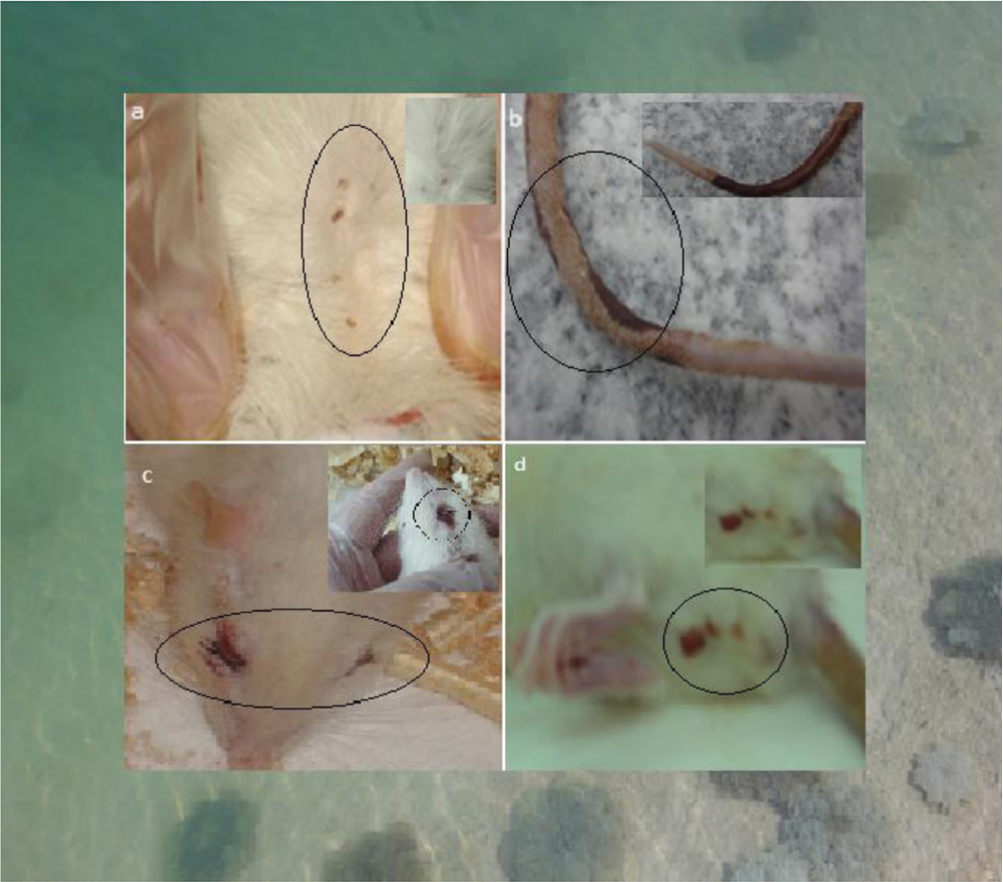
Scab-like spots on the skin of testing rats groups (a), and dermonecrosis in injection site (b), rectal bleeding (c) and eye bleeding (d), after 21 days exposure to *C. andromeda* jellyfish venom.

In addition to some general behaviors and signs such as severe pain feel, agitation and confusion, aggression, violence and libido sensation behaviors after injection [2], the dermonecrosis in the injection site, and the scab-like spots on the skin of the tested rats were perceived after 21 days exposure to different doses of the jellyfish venom. Rectal and eye bleeding (in corner of eyes) were also seen (Fig. 1).

## 2 Experimental design, materials and methods

All specimens of *C. andromeda* were collected from the Nayband bay, in the North (27° 30´ S, 52° 35´ E) of Bushehr-Iran [1], [2]. Nematocysts and tentacles were separated as described by Bloom et al. [3]. The mortality rate was measured within 24 hours, according to Wiltshire et al. [4]. For hematological parameters *in vivo*, eighteen male Wistar rats, (Weighing approximately 200±10 g), were distributed randomly among three groups (two separated groups for test and the other group for control (*n*=6)). Animals in two tested groups were given two dilution series of 1/2 and 1/3 LD50 of jellyfish venom, respectively. The same volume of distilled water was injected into the other group as negative control. After 12 h, the animals were anaesthetized by diethyl ether. Blood sample of each animal was collected by cardiac punctures, into both heparinized tubes for directly hematological tests, and without anticoagulant tubes for biochemical examinations. The performance evaluations of hematological parameters include RBC, WBC, Hb, MCV, MCH, MCHC and HCT were performed using the Sysmex XE-5000 hematology analyzer. All tests were done in triplicate and data were expressed as mean±SD. Statistical analysis was carried out using Kruskal–Wallis test. *P*<0.05 is considered statistically significant. The hemolysis experiment of crude venom was assessed using human erythrocytes according to the method of Garnier et al. [5].

For chemical analysis of serum electrolytes, a portion of free anticoagulant blood samples, which previously described, were centrifuged at 600×*g* for 15 minutes, and the supernatant was separated as the serum. Serum albumin, urea, creatinine, uric acid, glucose, triglyceride, and haptoglobin were also measured.

## Funding sources

The source of data used in this paper was the PhD thesis of Gholamhossein Mohebbi, student of the Bushehr University of Medical Sciences, and was funded by the Vice Chancellor of Research, Bushehr University of Medical Sciences, Bushehr-Iran.

## Figures and Tables

**Figure:**
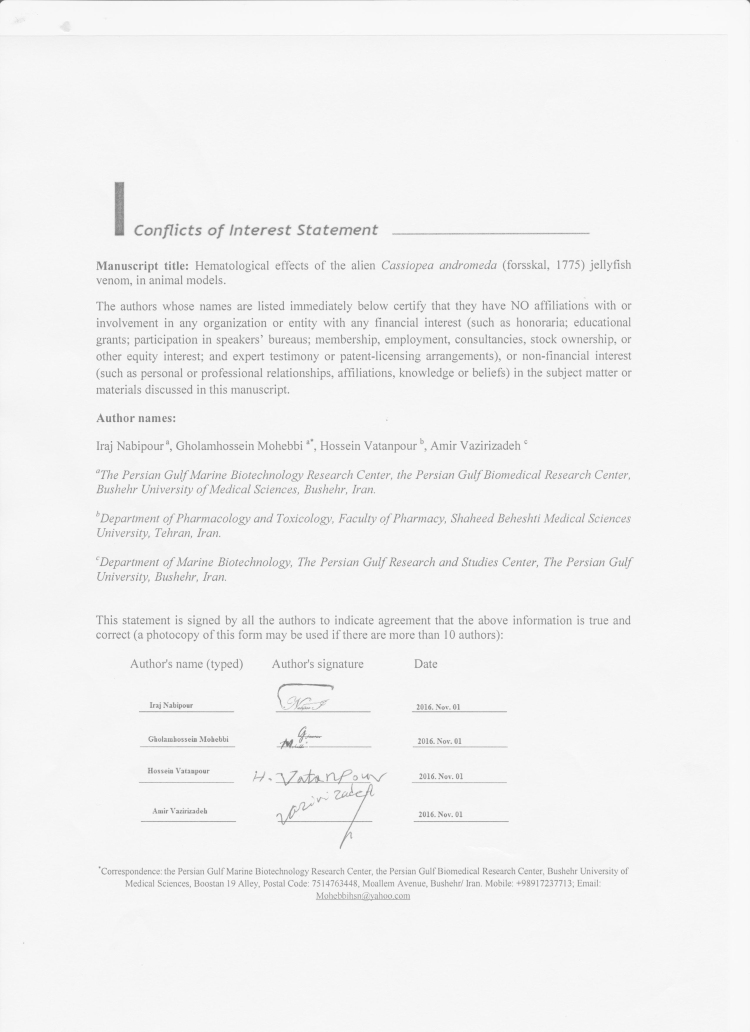
Supplementary material.

**Table 1:** The significant changes in the some hematological parameters and electrolyte levels of rat blood samples, after administration of two dilutions of 1/2 and 1/3 LD_50_ doses of jellyfish venom, compared with control group. All assays were carried out in triplicate.

Parameter	Group		
	Control (n:6)	1/3 LD_50_ (n:6)	½ LD_50_ (n:6)
RBC (Million/mm^3^)	3.46±0.017	2.9±0.01⁎	2.61±0.006⁎
WBC (Million/mm^3^)	4.37±0.029	5.53±0.012⁎	6.72±0.02⁎
Hb (g/dl)	16.29±0.032	7.44±0.042⁎	5.4±0.285⁎
MCV (fl)	56.71±0.38	40.51±0.005⁎	35.61±0.002⁎
MCH (pg)	18.72±0.061	16.76±0.028⁎	15.49±0.34⁎
MCHC (g/dl)	35.66±0.078	29.21±0.15⁎	32.44±0.005⁎
HCT (%)	44.3±0.006	25.37±0.05⁎	22.7±0.005⁎
Na (mg*/*dL*)*	155.47±0.51	149.99±0.09⁎⁎	144.19±2.038⁎⁎
K (mg/dL)	7.74±0.056	12.41±0.15⁎⁎	18.66±0.5⁎⁎
Ca (mg/dL)	13.19±0.16	23.03±0.05⁎⁎	28.39±0.61⁎⁎
Mg (mg/dL)	18.1±0.05	16.23±0.25⁎⁎	13.94±0.07⁎⁎
P (mg/dL)	53.1±0.11	75.07±0.11⁎⁎	102.53±1.45⁎⁎
Fe (mg/dL)	68.53±0.45	55.64±0.54⁎⁎	44.08±0.13⁎⁎
Albumin (g/L)	34.93±0.404	34.66±0.61	32.01±0.49⁎⁎
Creatinine (μmol/)	31.76±0.77	35.4±1.15⁎⁎	45.2±0.26⁎⁎
Uric Acid (μmol/)	155.05±0.48	76.01±1.73⁎⁎	65.69±0.36⁎⁎
Glucose (mmol/L)	4.86±0.17	4±0.02⁎⁎	2.36±0.07⁎⁎
Cholesterol (mmol/L)	1.49±0.03	1.63±0.002⁎	1.9±0.04⁎⁎
Triglyceride (mmol/L)	0.66±0.014	0.64±0.01	0.6±0.002⁎
Haptoglobin (mg/dL)	52.85±0.3	188.01±0.01⁎⁎	266.37±1.57⁎⁎

⁎ *p*<0.05 compared with control.

⁎⁎ *p*<0.005 compared with control.
